# Vaccine hesitancy among parents of children in their first two years of life

**DOI:** 10.3389/fpubh.2024.1438737

**Published:** 2024-09-19

**Authors:** Ruth Magyar, Peter K. Voitl, Julian J. M. Voitl, Susanne C. Diesner-Treiber

**Affiliations:** ^1^Outpatient Department, First Vienna Pediatric Medical Center, Vienna, Austria; ^2^Department of Pediatrics and Adolescent Medicine, Medical University of Vienna, Vienna, Austria

**Keywords:** adherence, vaccination, children, COVID-19, Austria, preterm birth

## Abstract

**Background:**

Vaccine hesitancy is considered a primary cause of outbreaks of vaccine-preventable infectious diseases. The Austrian vaccination plan includes 24 vaccinations in the first 2 years of life, 12 for free and 12 subject to a fee. Since preterm babies are more susceptible to severe infections, immunization is a vital protection strategy. This study examines the routine immunization schedule recommended for children in Austria, the number of timely vaccinations, and the number of delayed and rejected vaccinations. Possible reasons for vaccination delays and rejection and potential influencing factors (preterm birth, COVID-19 pandemic, information sources) are also analyzed.

**Methods:**

We included children aged 2 to 5 years who presented to Vienna's largest pediatric center with an Austrian mother-child pass and spent the first 2 years of their lives in Austria. Data was collected using questionnaires about the vaccination status, parents' reasons for any rejections or delays in the recommended vaccination regimen, the impact of the COVID-19 pandemic on individuals' vaccination behavior, and child-specific influencing factors such as preterm birth and socioeconomic factors.

**Results:**

90% of the 150 study subjects follow the recommendations on routine vaccinations, while 40–62% accept vaccinations subject to a fee. Preterm infants received less fee-based (53%) as well as gratuitous (88%) vaccinations. While free vaccinations tend to be delayed, more fee based vaccinations are rejected. With free vaccinations, delays and refusals occur due to illness or missed appointments. In the case of fee- required vaccinations, however, fears of side effects are also one of the main reasons. Due to the COVID-19 pandemic, about a quarter of parents have become more skeptical about vaccines. However, the vaccination rate of premature babies is usually just below that of full-term babies. Physicians remain the most trustworthy source of information about vaccinations.

**Conclusion:**

Free vaccinations are more accepted by parents than fee based vaccinations. Preterm babies, which are a high risk group for vaccination preventable diseases, show a lower or delayed vaccination rate, which must be prevented through intensive doctor education. In addition, vaccination hesitancy changed during the COVID-19 pandemic, which needs to be addressed during the medical consultation.

## Introduction

Child mortality has fallen sharply worldwide in recent decades thanks to vaccination campaigns (1, 2). Since the introduction of the WHO Expanded Program on Immunization (EPI) in 1974, vaccination was estimated to be directly responsible for 40% of reduced global infant mortality (3). However, increased negative attitudes toward vaccinations lead to a recurrence of vaccine-preventable diseases (4, 5). The 2023 Austrian vaccination plan recommends 22 vaccinations for children in the first 24 months of life. The state fully covers twelve of these; the others are subject to a fee. The free vaccinations include up to three partial vaccinations against rotavirus, three against diphtheria-tetanus-pertussis-poliomyelitis-Haemophilus influenzae B-hepatitis B (hexavalent), three against pneumococci (PCV), and two against measles-mumps-rubella (MMR). The following are part of the pay-for vaccination program: three vaccinations against meningococcal B, one against meningococcal ACW_135_Y, three against tick-borne encephalitis (TBE), two against varicella, and two against hepatitis A, the latter being only a travel immunization for high risk countries (6). According to the Austrian Ministry of Social Affairs, vaccination rates of those fully immunized against MMR and the hexavalent vaccine reached 87% in 2022, which is well below the recommended 95% limit recommended by the world health organization (WHO) (7, 8). Some population groups are at particular risk to develop infections and should preferably be immunized quickly. Premature babies have an above-average mortality rate due to their naive immune system and reduced diaplacental antibody transfer, making them more exposed to life-threatening infectious diseases (9, 10). According to the WHO, 15 million children are born before the 37th week of pregnancy every year (11). In 2022, the premature birth rate in Austria was 6.9% (12). Refusal of vaccination is primarily motivated by skepticism among parents and medical staff who fear side effects in such a vulnerable cohort, but delays might also be caused by hospital stays or scheduling conflicts (13). The COVID-19 pandemic has strongly impacted almost all areas of human life. The hard-fought progress in improving vaccination adherence over the last few decades has faced considerable setbacks, indicated by increasing numbers of measles or pertussis worldwide (14–16). According to WHO and UNICEF data this was the largest decline in childhood vaccinations in 30 years. In specific, diphtheria, tetanus and pertussis vaccine coverage dropped 5% points in 2021; the first dose of measles coverage has fallen more than 7% worldwide to 81% during the pandemic, being the lowest levels since 2008 (17, 18), which means that children are at higher risk for vaccine-preventable diseases.

The aim of this study was to assess parents' adherence to the Austrian vaccination recommendations of their children within the first 2 years of life.

Therefore, the number of timely vaccinations, those booked slightly late (1–3 months), the number of appointments booked with a significant delay (>3 months), and the number of refused vaccinations were investigated. Furthermore, possible reasons for vaccination delays and rejections are explored, as well as their connection to preterm birth, the COVID-19 pandemic, and sources of information about vaccinations.

## Material and methods

### Study design

This retrospective cross-sectional study was conducted at the “First Vienna Pediatric Medical Center” from January to February, 2023. Over 57,000 patients are seen at this center annually, or 16% of all outpatient care in Vienna. It is, therefore, the largest primary care facility in Austria (as of 2015) (19).

### Study population

Children aged 2 to 5 years who had an Austrian mother-child pass and spent the first 2 years of life in Austria were included. For better comparability, children who did not undergo the routine mother child pass examinations at the study center were excluded.

The Ethics Committee of the Medical University of Vienna (EK No. 1964/2023) approved this study. The legal guardians were informed about the study and had to give written consent.

The study involved a questionnaire (Supplementary Table 1) with general information about the participant (age, gender, preterm birth) and socioeconomic factors (father's/mother's age at birth, current father's/mother's age, highest educational qualification, number of siblings). Further information was collected regarding the accuracy, timeliness, and completeness of the vaccinations according to the Austrian vaccination recommendations in the first 2 years of life and information about the effects of the COVID-19 pandemic on vaccination behavior. To increase data accuracy, the study team checked the vaccination times from the vaccination certificate, calculated the age in months and compared them with the recommended vaccination time intervals. The vaccination appointments and intervals based on the recommendations of the Austrian vaccination schedule were examined and compared to determine minor (one to 3 months) or significant (>3 months) delays, as well as rejections. Respondents were asked to provide their motivations for delayed or rejected vaccinations. The vaccinations against rotavirus, pneumococcus, MMR, the hexavalent vaccine, and the first two doses against meningococcal B were considered delayed if administered at least 1 month later than the recommended schedule, which then was further divided into minor delays (1–3 months late) and significant delays (>3 months late). The vaccinations against meningococcal ACW_135_Y, TBE, hepatitis A, varicella, and the third meningococcal B dose were considered delayed if administered after the age of two (second birthday).

Data regarding the source of information about vaccinations and the impact of the COVID-19 pandemic on vaccination behavior were then analyzed.

### Statistics

The data collected in this study was entered as raw data in an Excel file and analyzed using IBM SPSS Statistics Version 29.0 software. The statistical significance level was set at p < 0.05. As this study was conducted over a fixed period of time (2 months), it was the aim to include at least 150 children (N). This number was set in consultation with a statistician in order to be able to represent a representative collective in this descriptive study over a fixed observation time. For this reason, a power analysis was not performed.

The nominal variable timing of vaccinations was divided into four groups: on time, 1–3 months late, more than 3 months late (summarized as vaccinated) and not vaccinated, which also resulted in the vaccination coverage rates. Other nominal variables were gender, preterm birth, reasons for vaccination delays/refusals, source of information regarding vaccinations, changes in vaccination behavior due to the Covid-19 pandemic and reasons for changes in vaccination behavior due to the Covid-19 pandemic. The nominal as well as the ordinal variable highest educational qualification were each given in absolute and relative frequencies (in %). For most metric data (father's age at birth, mother's age at birth, father's current age, mother's current age and number of siblings), the mean value and the 1st and 3rd quartiles were given for the number of valid values (n). The data for the metric variable age of the child, on the other hand, was determined with minimum and maximum. The descriptive statistics of the patient collective were presented using absolute and relative frequencies, the arithmetic mean and the interquartile range. Furthermore, the vaccination coverage rates (fully and partially immunized) as well as the timing of all vaccinations (total of all vaccinations, individual vaccines, free and paid vaccinations) were presented with absolute and relative frequencies. All reasons for vaccination delays and refusals (total of all vaccination delays and refusals, individual vaccines, free and paid vaccines) were also presented, as well as the data on the impact of the Covid-19 pandemic on vaccination behavior and the reasons for the change in vaccination behavior during the Covid-19 pandemic.

A chi-square test (χ^2^) was used to examine the categorical variables premature birth and vaccination delays or refusals (on time, 1–3 months late, >3 months late and not vaccinated) in total and in subdivision of the individual vaccinations as well as the paid and free vaccinations. The connection between the source of information regarding vaccinations and vaccination delays or refusals was analyzed. To check the effect size of the chi-square tests, Cramer's V was used on the basis of the classification according to Rea and Parker (*r* = 0.10–0.20 weak correlation, *r* = 0.20–0.40 moderate correlation and *r* = 0.40–0.60 relatively strong correlation) (20). The Bonferroni correction was applied to avoid errors due to multiple testing (21).

## Results

One hundred fifty children between the ages of two (after 2nd birthday) and five (before 5th birthday) who spent their first 2 years in Austria and had an Austrian mother child pass issued by the “First Vienna Pediatric Medical Center” were included in this study during the observation period from January to February, 2023. Among our study cohort, 48.7% were male, and 51.3% were female; 17.3% were born before the 37th week of pregnancy (Supplementary Table 2).

### General clinical data

According to the Austrian vaccination plan, timely vaccinations are administered within the recommended month. Minor delays refer to “up to 3 months,” and significant delays indicate “>3 months”. In total, 22.6% of the recommended vaccinations were not administered (Table 1), while 77.4% (*n* = 2,496) vaccines were administered either on time or delayed. Overall, vaccinations against rotavirus (48.8%) and against TBE (56.4%) and MMR (40.7%) were most often administered on time. The hexavalent vaccination (64.0%), and the pneumococcus (PCV) (65.1%) were most frequently performed with a delay of one to three months.

**Table 1:** Time of vaccination.

**Vaccinations**	**Time of vaccination**	**N**	**%**
Total vaccinations (*n* = 3,223)	Timely vaccination	551	30.4
	1–3 months delay	981	29.9
	>3 months delay	964	17.1
	Not vaccinated	551	22.6
**Specific vaccinations**			
Rotavirus (*n* = 373)	Timely vaccination	182	48.8
	1–3 months delay	163	43.7
	>3 months delay	6	1.6
	Not vaccinated	22	5.9
Diphtheria, tetanus, pertussis (whooping cough), polio, hepatitis B and Haemophilus influenzae type b (Hib) (*n* = 450)	Timely vaccination	35	7.8
	1–3 months delay	288	64.0
	>3 months delay	115	25.5
	Not vaccinated	12	2.7
Pneumococci (*n* = 450)	Timely vaccination	28	6.3
	1–3 months delay	293	65.1
	>3 months delay	105	23.3
	Not vaccinated	24	5.3
Measles-mumps-rubella (*n* = 300)	Timely vaccination	122	40.7
	1–3 months delay	83	27.7
	>3 months delay	78	26.0
	Not vaccinated	17	5.6
Meningococcal B (*n* = 450)	Timely vaccination	90	20.0
	1–3 months delay	86	19.1
	>3 months delay	139	30.9
	Not vaccinated	135	30.0
Meningococcal ACW_135_Y (*n* = 150)	Timely vaccination	62	41.3
	1–3 months delay	5	3.3
	>3 months delay	3	2.0
	Not vaccinated	80	53.4
Tick-borne encephalitis (*n* = 450)	Timely vaccination	254	56.4
	1–3 months delay	22	4.9
	>3 months delay	39	8.7
	Not vaccinated	135	30.0
Hepatitis A (*n* = 300)	Timely vaccination	101	33.7
	1–3 months delay	15	5.0
	>3 months delay	34	11.3
	Not vaccinated	150	50.0
Varicella (*n* = 300)	Timely vaccination	107	35.7
	1–3 months delay	9	3.0
	>3 months delay	32	10.7
	Not vaccinated	152	50.6

Data in absolute and relative frequencies (%) in relation to the number of administered vaccinations. N (free vaccines) = 1,573 (white), N (fee based vaccines) =1,650 (gray).

In contrast, patients received vaccinations against meningococcal B (30.9%) with >3 months delay. Vaccinations against meningococcal ACW_135_Y (53.4%), hepatitis A (50.0%), and varicella (50.6%) were the most refused (Table 1).

### Vaccination rate for the entire patient population

Complete vaccination coverage means all doses were administered; partial signifies one or more doses were not administered. The patient is considered not immunized in case of failure to administer even a single dose. We observed high complete vaccination rates (above 90%) for the cost-free vaccinations (rotavirus, hexavalent, PCV and MMR). In the case of more fee based vaccinations, however, the vaccination rates of those who are fully immunized ranged between 40% and 62% (Table 2).

**Table 2:** Vaccination rates of the entire cohort.

**Vaccination**	**Completely immunized**		**Partially immunized**		**Not immunized**	
	***N***	**%**	***N***	**%**	***N***	**%**
Rotavirus (*n* = 150)	142	94.7	1	0.7	7	4.6
Diphtheria, tetanus, pertussis (whooping cough), polio, hepatitis B and Haemophilus influenzae type B (*n* = 150)	142	94.7	6	4.0	2	1.3
Pneumococci (*n* = 150)	138	92.0	8	5.3	4	2.7
Measles-mumps-rubella (*n* = 150)	139	92.7	5	3.3	6	4.0
Meningococci B (*n* = 150)	93	62.0	21	14.0	36	24.0
Meningococci ACW_135_Y (*n* = 150)	70	46.7	0	0.0	80	53.3
Tick-borne encephalitis (*n* = 150)	70	46.7	54	36.0	26	17.3
Hepatitis A (*n* = 150)	60	40.0	30	20.0	60	40.0
Varicella (*n* = 150)	71	47.3	6	4.0	73	48.7

Data in absolute and relative frequencies (%) in relation to the specific vaccine. Free vaccine: white. Fee based vaccine: gray.

### Effect of preterm birth on vaccination adherence

A connection between premature births and rejected vaccinations was observed, with 29.3% of all vaccinations being rejected for premature babies and only 21.1% in full-term babies (Cramer's V 0.075/*p* < 0.001/p-adj. = 0.006) (Supplementary Table 3).

Vaccinations against rotavirus (41.5%) and TBE (48.7%) were most frequently administered on time to premature babies. The hexavalent vaccination (48.7%), the one against PCV (52.6%), and MMR vaccines (34.6%) were administered 1–3 months late in most cases. Vaccinations against meningococcal B (30.8%) were most frequently delayed by >3 months. In contrast, vaccinations against meningococcal ACW_135_Y (53.8%), hepatitis A (53.8%), and varicella (53.8%) were mostly rejected (Supplementary Table 3).

Among the fully immunized pre-term children, the vaccination coverage is between 77.0% and 88.5% (free vaccinations), while the fee based vaccinations rate falls between 34.6% and 53.8%. 96.0% of the full-term babies, but only 88.5% of the premature babies, received all hexavalent vaccinations (Cramer's V 0.254/p = 0.008/p-adj. 0 0083). 95.2% of full-term infants are completely immunized against PCV, while only 4.0% remain partially immunized. In comparison, 77.0% of premature infants receive total immunization and 11.5% partial immunization against PCV (Cramer's V 0.287/*p* = 0.002/p-adj. 0 0083) (Table 3).

**Table 3:** Vaccination rates, preterm/term babies.

**Vaccination**	**Preterm/term**	**Immunization status**	**Data**		**Cramer's V**	***p*-value**	***p*-adj**.
			***N***	**%**			
Rotavirus	Preterm babies (*n* = 26)	Complete immunization	23	88.5	/	0.172	/
		Partial immunization	0	0.0			
		No immunization	3	11.5			
	Full term babies (*n* = 124)	Complete immunization	119	96.0			
		Partial immunization	1	0.8			
		No immunization	4	3.2			
Diphtheria, tetanus, pertussis (whooping cough), polio, hepatitis B and Haemophilus influenzae typeB	Preterm babies (*n* = 26)	Complete immunization	23	88.5	0.254	0.008	0.0083
		Partial immunization	1	3.8			
		No immunization	2	7.7			
	Full term babies (*n* = 124)	Complete immunization	119	96.0			
		Partial immunization	5	4.0			
		No immunization	0	0.0			
Pneumococci	Preterm babies (*n* = 26)	Complete immunization	20	77.0	0.287	0.002	0.0083
		Partial immunization	3	11.5			
		No immunization	3	11.5			
	Full term babies (*n* = 124)	Complete immunization	118	95.2			
		Partial immunization	5	4.0			
		No immunization	1	0.8			
Measles-mumps-rubella	Preterm babies (*n* = 26)	Complete immunization	21	80.8	/	0.034	0.0083
		Partial immunization	2	7.7			
		No immunization	3	11.5			
	Term babies (*n* = 124)	Complete immunization	118	95.2			
	Partial immunization	3	2.4			
	No immunization	3	2.4			
Meningococci B	Preterm babies (*n* = 26)	Complete immunization	14	53.8	/	0.612	/
		Partial immunization	4	15.4			
		No immunization	8	30.8			
	Term babies (*n* = 124)	Complete immunization	79	63.7			
		Partial immunization	17	13.7			
		No immunization	28	22.6			
Meningococci C/ACW_135_Y	Preterm babies (*n* = 26)	Complete immunization	12	46.2	/	0.954	/
		Partial immunization	0	0.0			
		No immunization	14	53.8			
	Term babies (*n* = 124)	Complete immunization	58	46.8			
		Partial immunization	0	0.0			
		No immunization	66	53.2			
Tick-borne encephalitis	Preterm babies (*n* = 26)	Complete immunization	13	50.0	/	0.055	/
		Partial immunization	5	19.2			
		No immunization	8	30.8			
	Term babies (*n* = 124)	Complete immunization	57	46.0			
		Partial immunization	49	39.5			
		No immunization	18	14.5			
Hepatitis A	Preterm babies (*n* = 26)	Complete immunization	9	34.6	/	0.911	/
		Partial immunization	6	23.1			
		No immunization	11	42.3			
	Term babies (*n* = 124)	Complete immunization	51	41.1			
		Partial immunization	24	19.4			
		No immunization	49	39.5			
Varicella	Preterm babies (*n* = 26)	Complete immunization	12	46.2	/	0.487	/
		Partial immunization	0	0.0			
		No immunization	14	53.8			
	Term babies (*n* = 124)	Complete immunization	59	47.6			
		Partial immunization	6	4.8			
		No immunization	59	47.6			

Data in absolute and relative frequencies (%) in relation to the number of administered vaccinations. Free vaccines: white, fee based vaccines: gray. P-value, p-adjust (p-adj.) after Bonferroni correction, Cramer's V. Preterm n = 26, Term n = 124.

### Timely administration of vaccinations

As for the free vaccines, 21 children (14.0%) of the entire cohort (*n* = 150) received all shots against rotavirus, one child received all partial vaccinations against PCV (0.7%), and 29 children (19.3%) were both times vaccinated against MMR on time. No child (0.0%) received all hexavalent vaccinations on time. In contrast, as for the fee based vaccinations, four children (2.7%) received all vaccinations against meningococcal B, 62 children (42.3%) were completely vaccinated against meningococcal ACW_135_Y, 23 children (15.3%) received all partial vaccinations against TBE, 29 Children (19.3%) got both vaccinations against hepatitis A, and 49 children (32.7%) were administered both partial vaccinations against varicella on time.

### Reasons for vaccination delays and refusal

The main reasons for vaccination delays and refusal include “missed appointment” (28.1%) and “child illness” (36.4%). However, other factors, such as “fear of side effects” (20.9%), “financial reasons” (1.7%), or “vaccination is not free and therefore not essential” (2.4%), also play a role (Table 4).

**Table 4:** Reasons for delays/Vaccination behavior change caused by the COVID-19 pandemic/Source of information.

**I. Reasons for delays and refusals**						
					***N***	**%**
Reasons for delays and refusals (*n* = 2,242)	Missed appointment				631	28.1
	Child illness				817	36.4
	Fear of side effects				468	20.9
	Congenital disease				17	0.8
	Financial reasons				38	1.7
	Vaccination is not free of charge and therefore not necessary				53	2.4
	Child should heal itself				36	1.6
	Child already had varicella				7	0.3
	Other				175	7.8
Data in absolute and relative frequencies (%) in relation to all the reasons for vaccination delays and refusals						
**II. Vaccination behavior change caused by the COVID-19 pandemic (*****n*** = **150)**						
					***N***	**%**
yes					40	26.7
no					110	73.3
**III. Reasons behind the vaccination behavior change (*****n*** = **40)**						
					***N***	**%**
Vaccines are important to me					7	17.5
I am more skeptical					25	62.5
Vaccination appointment missed due to the pandemic					2	5.0
Fear of attending the appointment due to COVID-19					6	15.0
Data in absolute and relative frequencies (%) in relation to the reasons for vaccination behavior change						
**IV. Source of information for timely, 1–3 months delay**, >**3 months delay and refused vaccines**						
**Information source**	**Vaccination time**	**Data**		**Cramer's V**	***p*** **-value**	***p*** **-adj**
		***N***	**%**			
Doctor (*n* = 2,596)	Timely vaccination	845	32.6	0.102	< 0.001	0.0025
	1–3 months delay	794	30.6			
	>3 months delay	452	17.4			
	Not vaccinated	505	19.4			
Medical journals (*n* = 172)	Timely vaccination	35	20.2			
	1–3 months delay	37	21.5			
	>3 months delay	32	18.6			
	Not vaccinated	68	39.5			
Magazine (*n* = 22)	Timely vaccination	4	18.2			
	1–3 months delay	9	40.9			
	>3 months delay	7	31.8			
	Not vaccinated	2	9.1			
Website (*n* = 259)	Timely vaccination	66	25.5			
	1–3 months delay	75	29.0			
	>3 months delay	32	12.3			
	Not vaccinated	86	33.2			
Social Media (*n* = 174)	Timely vaccination	31	17.8			
	1–3 months delay	49	28.2			
	>3 months delay	28	16.1			
	Not vaccinated	66	37.9			

Data in absolute and relative frequencies (%) in relation to the information source.

P-value, p-adjust (p-adj.) after Bonferroni correction, Cramer's V.

**Figure 1:**
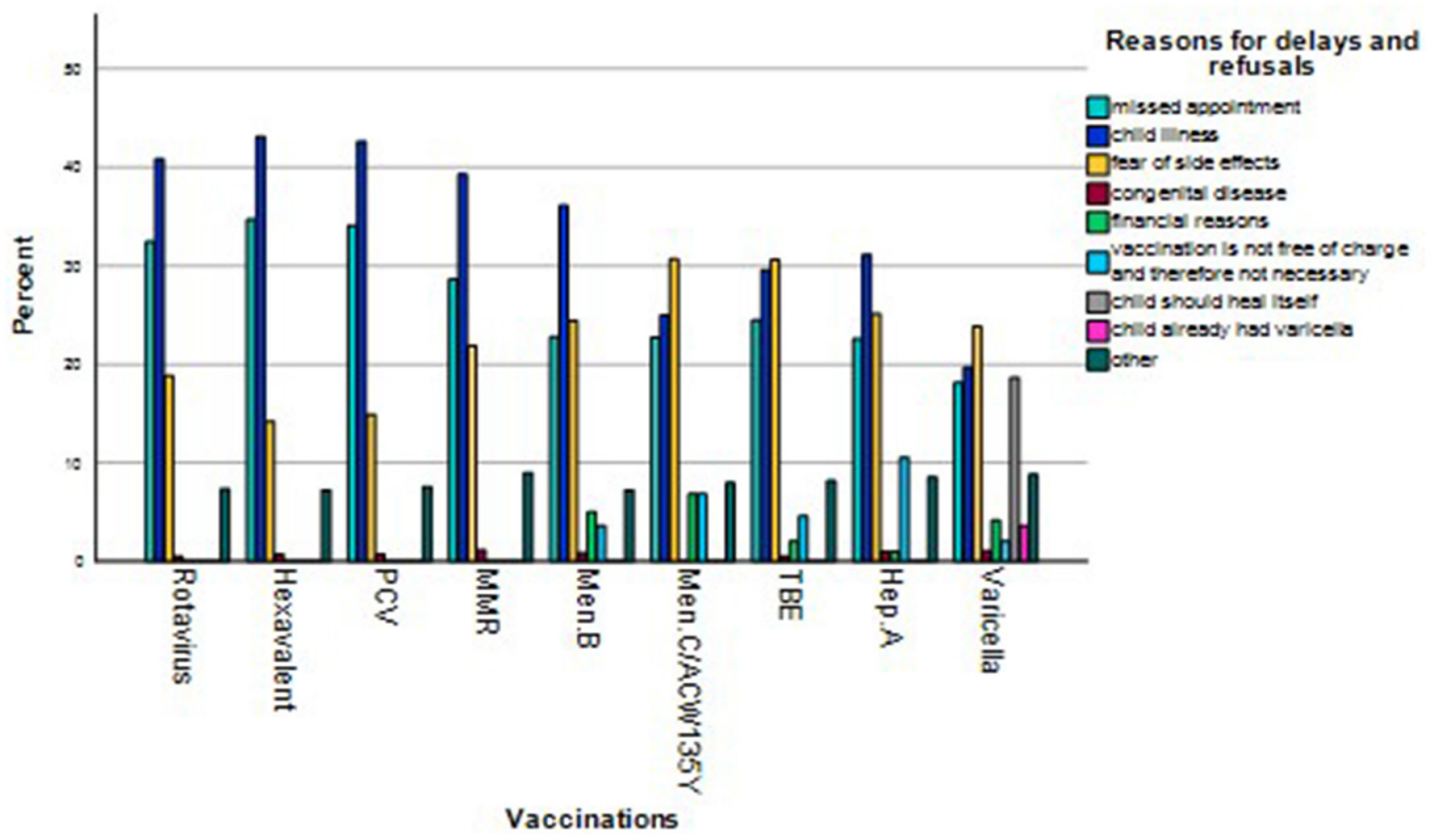
Reasons for delays and refusals.

While “missed appointment” and “child illness” were the most frequent reasons for vaccination delays and refusals for free vaccinations, “fear of side effects” is the prevailing answer for fee based vaccinations. However, in some cases, “financial reasons” and “vaccination is not free and therefore not essential” were also cited as reasons for delayed and refused vaccinations (Figure 1).

There is a strong correlation in the entire cohort between free or fee based vaccinations and timely, but also delayed or not carried out vaccinations, with 95.0% of the free vaccines but only 60.5% of the fee based ones being administered (Cramer's V 0.562/*p* < 0.001/*p*-adj. = 0.00625) (Table 5). While free vaccinations are more likely to be carried out late, fee based vaccinations are more likely to be refused. This finding also indicates a moderate correlation between free or fee based vaccinations and vaccinations carried out or refused in premature babies, with 97.5% of the free vaccinations and only 63.2% of fee based vaccinations administered to premature infants (Cramer's V 0.4/*p* < 0.001/*p*-adj. = 0.0083) (Table 5).

**Table 5:** Vaccination time for free and fee based vaccinations of the entire cohort/Relationship between refusals in preterm babies and free or fee based vaccinations.

**Vaccinations**	**Vaccination time**	**Data**		***p*-value**	***p*-adj**.	**Cramer's V**
		**N**	**%**			
All free vaccinations (*n* = 1573) 1,498 vaccinated (95.2%)	On time	367	23.3	< 0.001	0.00625	0.562
	1–3 months delay	827	52.6			
	>3 months delay	304	19.3			
	Not vaccinated	75	4.8			
All fee based vaccinations (*n* = 1,650) 998 vaccinated (60.5%)	On time	614	37.2			
	1–3 months delay	137	8.3			
	>3 months delay	247	15.0			
	Not vaccinated	652	39.5			
**Relationship between vaccination refusals in preterm babies and free or fee based vaccines**						
**Vaccination rates**	**vaccinated/not vaccinated**	**Data**		***p*** **-value**	***p*****-adj**.	**Cramer's V**
		***N***	**%**			
Vaccination rate–free vaccines	Vaccinated	581	97.5	< 0.001	0.0083	0.40
	Not vaccinated	19	2.5			
Vaccination rate–fee based vaccines	Vaccinated	475	63.2			
	Not vaccinated	275	36.8			

Data in absolute and relative frequencies (%) related to the vaccination rates for free and pay-for vaccines.

P-value, p-adjust (p-adj.) after Bonferroni correction, Cramer's V.

### Impact of the COVID-19 pandemic

Of the legal guardians who reported that the COVID-19 pandemic changed their vaccination attitude, over 26% became more hesitant. For some, however, the pandemic has strengthened their pro-vaccination stance (Table 4).

### Source of information

Physicians are the most reliable advisors regarding vaccinations. Therefore, consultations appear connected to the percentage of timely and refused vaccinations, with 32.6% of timely vaccinations and only 19.4% rejected appointments.

The role of social media in conveying information regarding vaccinations is also fundamental (Cramer's V 0.102/*p* < 0.001/*p*-adj. = 0.0025) (Table 4).

## Discussion

While the vaccination rate among fully immunized children is over 90% when it comes to free vaccinations, that of fee based vaccinations fluctuates between 40% and 62%. While free vaccinations tend to be performed late, fee based vaccinations are more likely to be refused, which results in a moderate correlation between free or fee based vaccinations and administered or refused ones. Furthermore, a strong correlation emerges between free or fee based vaccinations and vaccinations administered on time, with a delay, or not at all. A comparison between the vaccination rates among premature and full-term infants proves that the first group is typically less likely to be vaccinated than the second one. Vaccines against meningococcal ACW_135_Y, hepatitis A, and varicella are most frequently refused, both among premature babies and the entire patient population. There is a slight correlation between preterm birth and lack of immunization when it comes to free vaccinations. In the case of fee based vaccinations, however, there is no connection between prematurity and vaccination delays/rejections. The main reasons behind delays and refusals include child illness and missed appointments. However, the fear of side effects is also common for fee based vaccinations, followed by financial reasons and the assumption that the vaccines that come for a cost are negligible. For around a quarter of legal guardians, vaccination behavior has changed so considerably due to the COVID-19 pandemic that some have become highly skeptical.

Doctors are the most influential information providers when it comes to vaccines. There is a slight connection between information providers and timely and rejected vaccinations.

We categorized delayed vaccinations for those vaccines being recommended in the first year of life into minor delays (1–3 months) and significant delays (>3 months). Other studies have also defined 1 month late as a delay in vaccination schedules (22). It is believed that this delay should not put the child at any significant risk. Prior to this study, we have observed that short delays of a few weeks might mainly be due to infections or problems making an appointment. However, longer delays seem to be associated with skepticism, which is why we wanted to analyze differences with this classification. For the vaccines recommended after the first birthday a delay was defined as vaccination after the age of two. This is because in Austria a number of vaccinations are recommended in the second year of life without any clear ranking. A ranking is then also based on seasonality, e.g. tick vaccination in spring and summer.

This hypothesis was in line with our results, as delays < 3 months are often due to financial reasons or increased infection rates in the winter months, also among premature babies. In this case, scheduling conflicts seem to play a significant role. Delays >3 months are primarily motivated by skepticism.

In this study, the vaccination rate of those fully immunized against rotavirus was 94.7%, against 80% reported in international studies (23, 24). For vaccinations against MMR, the percentage was 92.7%, compared to 78.3% in Germany and Austria (25), 78% in the Netherlands (26), and 94.3% in the USA (27). The vaccination rates against meningococcal B and TBE in this study reached 62.0% and 46.7%, respectively, higher than those observed abroad (28.9%, 10%) (28–30). The vaccination rate of the fully immunized against varicella in this study is 47.3%. Research efforts in Poland and Sweden document that varicella vaccinations are not part of the national routine vaccination schedule, and vaccination rates only reach 4.2% and 15%, respectively (31). In general, in Austria, vaccinations are not mandatory (6).

Since premature babies are immunologically immature, they are at exceptionally high risk for infectious diseases (13), and their need for increased medical attention may increase the risk of infections (32); early and complete immunization is essential in this patient group (33). The vaccination rate of fully immunized premature infants observed in this study is 88.5% for hexavalent vaccinations and 76.9% against PCV. A German study, however, describes that only 47% of premature babies received all vaccines against pneumococci, while 71% had received all hexavalent doses. However, premature babies were vaccinated according to the 3+1 vaccination schedule (34). Contrarily, our cohort's immunization routine followed the 2+1 system.

Austria is in part critical toward vaccination recommendations. Especially during the COVID-19 pandemic, the population and parents were confronted with false information in social media by so-called self-proclaimed experts. In a recent Austrian study, vaccine hesitancy was motivated by concerns about “personal freedom restriction”, “lack of trust in the pharmaceutical industry” and “lack of trust in the government” which were not receptive to discussion. However, concerns such as “side effects”, “long-term side effects” and “questionable benefit of vaccination” could be ruled out by evidence-based information. Parental vaccination hesitancy and social characteristics such as gender, educational level, economic status or political opinion are associated (35).

As mentioned, the COVID-19 pandemic sparked the vaccination debate worldwide. According to the WHO, the PCV vaccination rate plunged from 86% in 2019 to 83% the following year, or 22.7 million children who did not receive their vaccine doses (36), meaning preventive measures reduced vaccination rates. In contrast, our findings show a vaccination rate of 92% for those fully immunized against PCV. The correlation between timely vaccinations, refused vaccination, and legal guardians who receive information about vaccination from their physicians indicates that doctors' recommendations contribute greatly to both timely administration and refusals. A Danish study shows that positive attitudes toward MMR vaccination among general practitioners led to average vaccination rates of 85%, compared to 69% in practices with stronger hesitancy toward vaccination (37).

### Limitations

The data of children whose parents refused to participate could not be recorded. Additionally, children whose parents are totally against vaccines also refused to take part in the study, which indeed leads to a data bias. Another limiting factor is the monocentric study design. However, the “First Vienna Pediatric Medical Center” is the largest group practice in Austria, meaning our study population can be considered reasonably representative. In addition, “child illness” and “missed appointment” were mentioned frequently as reasons for vaccination delays and refusals, which may indicate a participant bias.

## Conclusion

In summary, vaccination coverage rates are quite remarkable in our study. These are over 90% for the free vaccinations and between 40% (hepatitis A) and 62.0% (meningococcal B) for the fee based vaccinations. There is a strong connection between free and administered vaccinations and between fee based and refused vaccinations, with 95.2% of the free vaccinations being accepted as opposed to only 60.5% of the fee based vaccinations. Except for TBE, the vaccination coverage of premature babies is below that of full-term babies. Approximately the same percentage of free and fee based vaccinations were administered on time. While free-of-charge vaccinations are more likely delayed in the entire patient population and in premature babies, fee based vaccinations are more likely rejected.

As for free vaccinations, we noticed a mild correlation between premature births and rejected vaccinations. While “child illness” and “missed appointment” are the most frequent reasons for vaccination delays and refusals for free vaccinations, “fear of side effects” is the most common for vaccinations that are not free of charge. In addition, the COVID-19 pandemic influenced legal guardians' vaccination behavior and increased their hesitancy. The importance attached to the role of the doctor can significantly influence the vaccination behavior of many. Considering these findings, information about vaccines could be more efficiently delivered, especially in relation to premature babies and specific fee based vaccinations.

## Data Availability

The original contributions presented in the study are included in the article/Supplementary material, further inquiries can be directed to the corresponding author.
