# Supplementary material for: Vaccine hesitancy among parents of children in their first two years of life
Source: Front Public Health. 2024 Sep 19;12:1438737. doi: 10.3389/fpubh.2024.1438737 (PMC11448122; doi:10.3389/fpubh.2024.1438737)
Supplement: Supplementary file 1 [file Table_1.docx]

| **Supplementary Table 1. Case Report** | | |
| --- | --- | --- |
| Date: ____________________ | | |
| Patient-ID: ____________________ | | |
| **Please answer the following questions for the child's father:** | | |
| Highest educational qualification? | ⭘ no graduation  ⭘ high school diploma  ⭘ general qualification for university entrance  ⭘ academic qualification | |
| Age at birth of child? | ­­­__________________ | |
| Current age? | __________________ | |
| **Please answer the following questions for the child's mother:** | | |
| Highest educational qualification? | ⭘ no school-leaving qualification  ⭘ high school diploma  ⭘ general qualification for university entrance  ⭘ academic qualification | |
| Age at birth of child? | ­­­__________________ | |
| Current age? | __________________ | |
| **Questions about your child:** | | |
| Gender: | ⭘ male | ⭘ female |
| Age of the child? | _________________ | |
| Number of siblings? | _________________ | |
| Was your child born before the 37th week of pregnancy? (premature baby) | ⭘ yes | ⭘ no |

| **Supplementary Table 2. Sociodemographics** | | | |
| --- | --- | --- | --- |
| **Variable** | **Categories** | **Data** | |
| **Age (in years)** |  | 2.8 | 2-5 |
| Sex | male | 73 | 48.7% |
|  | female | 77 | 51.3% |
| Siblings (number) |  | 0.76 | 0-3 |
| Age of father at child’s birth (in years) |  | 30.7 | 26-34.25 |
| Age of mother at child’s birth (in years) |  | 29.0 | 25-32 |
| Current age of father (in years) |  | 33.7 | 30-37 |
| Current age of mother (in years) |  | 32.0 | 29-35 |
| Highest parental education | none | 0 | 0 |
|  | secondary education | 63 | 42% |
|  | high school diploma | 34 | 22.7% |
|  | college degree | 53 | 35.3% |
| Preterm birth |  | 26 | 17.3% |
| Term birth |  | 124 | 82.7% |
| Data in absolute (left column) and relative frequencies (%, right column), arithmetic mean median (left column) and interquartile range (right column), N=150 | | | |

| **Supplementary Table 3.** **Time of vaccination in preterm babies** | | | | | | |
| --- | --- | --- | --- | --- | --- | --- |
| **Vaccinations** | **Time of vaccination** | **Data** | | **Cramer´s V** | **p-value** | **p-adj.** |
| **Total vaccinations** (n=559,  395 vaccinated (70.7%) | timely vaccination | 159 | 28.5 | 0.075 | p<0.001 | 0.00625 |
|  | 1-3 months delay | 146 | 26.1 |  |  |  |
|  | >3 months delay | 90 | 16.1 |  |  |  |
|  | not vaccinated | 164 | 29.3 |  |  |  |
| **Specific vaccinations** |  |  |  |  |  |  |
| Rotavirus  (n=65) | timely vaccination | 27 | 41.5 | 0.193 | 0.003 | 0.00625 |
|  | 1-3 months delay | 26 | 40.0 |  |  |  |
|  | >3 months delay | 3 | 4.6 |  |  |  |
|  | not vaccinated | 9 | 13.9 |  |  |  |
| Diphtheria, tetanus, pertussis (whooping cough), polio, hepatitis B and Haemophilus influenzae type b (Hib)  (n=78) | timely vaccination | 12 | 15.4 | 0.234 | <0.001 | 0.00625 |
|  | 1-3 months delay | 38 | 48.7 |  |  |  |
|  | >3 months delay | 21 | 26.9 |  |  |  |
|  | not vaccinated | 7 | 9.0 |  |  |  |
| Pneumococci  (n=78) | timely vaccination | 9 | 11.5 | 0.234 | <0.001 | 0.00625 |
|  | 1-3 months delay | 41 | 52.6 |  |  |  |
|  | >3 months delay | 16 | 20.5 |  |  |  |
|  | not vaccinated | 12 | 15.4 |  |  |  |
| Measles-mumps-rubella  (n=52) | timely vaccination | 15 | 28.8 | 0.234 | 0.003 | 0.00625 |
|  | 1-3 months delay | 18 | 34.6 |  |  |  |
|  | >3 months delay | 11 | 21.2 |  |  |  |
|  | not vaccinated | 8 | 15.4 |  |  |  |
| Meningococcal B  (n=78) | timely vaccination | 14 | 17.9 | / | 0.383 | / |
|  | 1-3 months delay | 11 | 14.1 |  |  |  |
|  | >3 months delay | 24 | 30.8 |  |  |  |
|  | not vaccinated | 29 | 37.2 |  |  |  |
| Meningococcal C/ACW_135_Y  (n=26) | timely vaccination | 11 | 42.3 | / | 0.661 | / |
|  | 1-3 months delay | 0 | 0.0 |  |  |  |
|  | >3 months delay | 1 | 3.9 |  |  |  |
|  | not vaccinated | 14 | 53.8 |  |  |  |
| Tick-borne encephalitis  (n=78) | timely vaccination | 38 | 48.7 | / | 0.366 | / |
|  | 1-3 months delay | 5 | 6.4 |  |  |  |
|  | >3 months delay | 6 | 7.7 |  |  |  |
|  | not vaccinated | 29 | 37.2 |  |  |  |
| Hepatitis A  (n=52) | timely vaccination | 17 | 32.7 | / | 0.798 | / |
|  | 1-3 months delay | 3 | 5.8 |  |  |  |
|  | >3 months delay | 4 | 7.7 |  |  |  |
|  | not vaccinated | 28 | 53.8 |  |  |  |
| Varicella  (n=52) | timely vaccination | 16 | 30.8 | / | 0.128 | / |
|  | 1-3 months delay | 4 | 7.7 |  |  |  |
|  | >3 months delay | 4 | 7.7 |  |  |  |
|  | not vaccinated | 28 | 53.8 |  |  |  |
| Data in absolute and relative frequencies (%) in relation to the number of administered vaccinations. Free vaccines: white, fee based vaccines: grey. P-value, p-adjust (p-adj.) after Bonferroni correction, Cramer’s V. | | | | | | |
